# Effect of Sex and Reproductive Status on Inhibitory Control and Social Cognition in the Domestic Dog (*Canis familiaris*)

**DOI:** 10.3390/ani11082448

**Published:** 2021-08-20

**Authors:** Saara Junttila, Salla Huohvanainen, Katriina Tiira

**Affiliations:** 1 Department of Production Animal Medicine, University of Helsinki, P.O. Box 57, 00014 Helsinki, Finland; 2 Department of Environmental and Biological Sciences, University of Eastern Finland, Yliopistokatu 7, 80101 Joensuu, Finland; salla.huohvanainen@gmail.com; 3 Department of Equine and Small Animal Medicine, University of Helsinki, P.O. Box 57, 00014 Yliopistokatu, Finland; katriina.tiira@helsinki.fi; 4 SmartDOG, Pietilänkatu 5, 11130 Riihimäki, Finland

**Keywords:** cognition, psychology, canine, animal behavior, cylinder test, unsolvable task, dog, neutering, sex differences, impulsivity, inhibitory control, social cognition

## Abstract

**Simple Summary:**

Various behavioral differences exist between male and female dogs, but very little research has focused on how sex influences cognition. Even fewer studies have taken sex hormones into account. Our aim was to investigate whether dogs’ sex and neutering status can influence two important cognitive traits: inhibitory control and social cognition. Inhibitory control was assessed using the cylinder test. In this task, the dog is required to inhibit reaching for a treat directly through a transparent barrier, and instead go around the barrier to access the treat. Social cognition was assessed using the unsolvable task, during which a food reward is visible but impossible to access. Dogs have three opportunities for action in this situation: (*a*) persisting with the problem independently, (*b*) seeking attention from a human, or (*c*) abandoning the task. Males were more impulsive and independent compared to females, whereas females had greater inhibitory control and were more likely to gaze at a human during a problem-solving situation. Since neutering status did not affect the results, it seems likely that these sex differences arose during early development and were not affected by levels of circulating sex hormones to a great extent.

**Abstract:**

Sex differences in a variety of cognitive traits have long been reported in various species, including dogs. However, only a few canine studies have taken the possible effect of reproductive hormones into account. The aim of this study was to investigate the effects of sex and reproductive status of pet dogs (*N* = 1032) on two cognitive traits: inhibitory control and social cognition. Inhibitory control was assessed using the cylinder test, and the dogs’ tendency to initiate social contact with a human during a problem-solving situation was assessed using the unsolvable task. Female dogs had a significantly higher success rate in the cylinder test compared to males, and they spent significantly more time in human-directed behavior during the unsolvable task. In contrast, males spent significantly more time in independent behavior during the unsolvable task. Reproductive status had no significant effect on the results of the cylinder test or the unsolvable task. Our results showed that female dogs asked for more help/used a more cooperative strategy during a problem-solving situation and had greater inhibitory control compared to males. According to our results, it seems likely that these sex differences were not influenced to a large extent by reproductive hormones.

## 1. Introduction

Sex differences have long been reported in various species for several cognitive abilities, such as spatial cognition [1,2] and learning ability [3]. Similar cognitive differences between males and females have also been found in dogs [4,5,6,7], but only a few studies have investigated the topic so far. These sex-specific behavioral differences are likely the result of evolutionary processes and may have arisen due to different selection pressures on males and females. Exploring the basis of these sex differences is of prime relevance for our understanding of canine cognition and behavior.

Sex differences may arise through three main mechanisms [8]: (*a*) genes located on sex chromosomes can cause differences in phenotype, (*b*) sex hormones may act on brain development before birth, which then leads to sex-specific traits arising later in life (organizational effect), or (*c*) different levels of circulating hormones in adulthood may cause differences between males and females (activational effect).

Inhibitory control is a cognitive skill which has been extensively researched in humans and other animals. It is defined as the ability to suppress an immediate, ineffective behavior in favor of a more advantageous one. In contrast, lack of inhibitory control—impulsivity—is often regarded as the tendency to act prematurely, without forethought or consideration for the consequences. In dogs, impulsivity has been shown to be a highly heritable trait [9], and it is associated with a greater likelihood of certain behavior problems (e.g., aggression) [10,11,12,13,14].

Impulsivity can be roughly divided into two broad concepts: impulsive action and impulsive choice [15]. Impulsive action refers to an individual’s inability to inhibit an immediate, counterproductive motor action in response to prepotent stimuli. Impulsive choice reflects an individual’s preference for a smaller, immediate reward over a delayed one of greater value or quantity.

The cylinder task is a behavioral test which has been used in a variety of species to measure impulsive action [16]. The subject is required to control its impulse to attempt to reach a visible food reward directly through a transparent barrier, and instead to go around the barrier to gain access to the food. MacLean and colleagues [16] tested 36 different species and found that absolute brain volume was associated with success on the cylinder task. The cylinder task has been used extensively in canine cognitive research [17,18,19,20]. However, no conclusion has been reached on the optimal method of measuring impulsivity in dogs [21,22]. We chose the cylinder test as it does not require a long/demanding training period. Any task that requires long training periods may actually exclude the most impulsive individuals, as poor impulse control most likely slows down the learning process. In addition, inhibitory control measured using the cylinder task has recently been found to be associated with working dog success [23].

In many species, especially mammals, impulsive action is often higher in males, whereas impulsive choice seems to be greater in females [24]. However, this seems to also depend on the task, species, and the reinforcer, since various studies have found opposing results or no sex differences at all [25]. Consistent with reports in many other mammals, female dogs seem to be more likely to make impulsive choices than males [26]. However, studies investigating impulsive action have found no sex differences in dogs [17,19,27], whether using the cylinder test or other tests. Fadel and colleagues [28] similarly found that sex had no effect on general impulsivity, when assessed using a validated, owner-filled questionnaire (Dog Impulsivity Assessment Scale, DIAS). However, a recent questionnaire study with a large sample size (13,700 dogs) found males to be more impulsive/hyperactive [29]. Refs. [17,19,26,27,28] In impulsivity studies that have taken reproductive hormones into account, the evidence on sex differences is more consistent, whereas studies that did not account for sex hormones have provided mixed results [24]. Testosterone levels seem to increase impulsive behavior in men [30], and gonadectomy has been shown to decrease impulsive action in male rats [31]. In female rats, gonadectomy was found to increase impulsive action [31], whereas progesterone treatment decreased it [32]. In dogs, neutered males seem to be more impulsive than intact males, according to a DIAS questionnaire [28]. This is in line with results in rats, which showed that gonadectomy increased impulsive choice in males [33].

Another extensively studied aspect of canine cognition is their social communicative ability in their interactions with humans. When dogs are incapable of solving a problem where food is visible but not accessible, they often seek human attention by using eye-contact or other human-directed social behaviors [34]. In contrast, wolves and other species tend to attempt to solve the problem independently, even when raised with humans from birth [35,36]. The dogs’ human-directed gazing behavior has been interpreted as a social problem-solving strategy, and as a request for help from the human [27,34,37,38]. This behavior shows individual and breed variation [39] and seems to also have a genetic component [40,41].

Two studies have reported that female dogs show more human-directed contact-seeking behavior during an unsolvable problem compared to males [40,41]. However, one study failed to replicate these findings [39], and another study found no sex differences in human-directed gazing during a solvable problem [42]. In general, female dogs spend more time gazing at their owner or engaging in social interactions with people in various situations [43,44,45,46,47]. In addition, visual information seems to be especially important to female dogs [48]. It is possible that reproductive hormones are responsible for some of these behavioral differences. Intact females spend more time looking at their owner compared to neutered females [47], and they are more likely to follow human visual signals than neutered females [49].

The first aim of this study was to investigate whether male and female dogs differ in their tendency for impulsive action in the cylinder test and in their contact-seeking behavior during an unsolvable task. Our second aim was to investigate whether reproductive status influences these traits. According to our knowledge, no studies have so far taken sex hormones into account or investigated whether neutering influences impulsive action or social behavior during an unsolvable task in dogs.

## 2. Materials and Methods

This study was approved by the Viikki Campus Research Ethics Committee of the University of Helsinki. All participating dog owners gave their written informed consent for inclusion of their dogs’ test results before they participated in the study.

### 2.1. Subjects

We used a dataset of 1332 Finnish privately owned dogs (483 males and 549 females) which participated in a commercial cognitive test battery (smartDOG™) [50]. The majority of subjects lived as pet dogs with their owners. No specific criteria for inclusion in the study were required, apart from the dogs being interested in working for treats and not being overly aggressive toward people. We limited the analysis to over 1 year old dogs, as the studied cognitive traits may still be developing in younger dogs [27,51]. 

After excluding dogs under 12 months, the ages of the dogs varied between 13 months and 14 years (Table 1), and they represented 131 breeds (Table A1). Three dogs had to be removed due to the sex of the dog missing from the records. Out of the remaining dogs, 23 individuals (16 male and seven female) did not complete either of the cognitive tests and were, therefore, excluded from the analysis. This resulted in a final sample size of 1032 dogs (46.8% male and 53.2% female) (Table 1). Seven dogs had no results for the cylinder task (three males and four females), and eight dogs had no results for the unsolvable task (one male and seven females). Reasons for not completing the tests included being afraid of the apparatus, not being motivated by the reward, or not passing the required criteria in the training phases. The completion rate of both tests was high (97%).

Reproductive status was not available for 485 dogs and, therefore, the sample size used for analyses involving reproductive status was 547 dogs. Of these, 28.3% were neutered and 71.7% were intact (Table 1).

### 2.2. Cognitive Test Battery

The data were obtained from results of a smartDOG™ cognitive test battery. SmartDOG™ is a company developed by Adj. Professor Katriina Tiira. Tests were performed by five trained smartDOG licence testers at testing sites across Finland, between 2016 and 2018. Owners cover their dogs’ testing expenses and receive a detailed report of their dogs’ cognitive performance, as well as access to a database where they can compare their dogs’ results to others.

The majority of dogs (77%) took part in a test battery called COGNITION, which lasts approximately 1.5 h and includes 11 tests measuring cognition, personality, and activity levels. A number of dogs (23%) took part in a shorter test which included 4–5 test parts and lasted approximately 1 h. All tests are based on previously published research, and they involve searching for food and solving various problems. Tests were performed in the same order for each dog, and the cylinder test was always performed first. In the current study, only results from the cylinder test and the unsolvable task were analyzed and discussed.

The test battery was performed indoors, in a room unfamiliar to the dog. Usually, only the dog, tester, and owner were present in the room, but other family members were occasionally present as observers. The dog was released upon entering the test room and allowed to explore the area freely for approximately 5 min, while the owner filled out the dog’s information sheet. The dog was off the lead for the duration of the test battery. The owner held the dog by its collar at the start of each trial, until the experimenter gave permission to release the dog. Food treats were used as rewards in each task, although occasionally a toy was used, if the dog was more motivated by toys than food. The type of treat used as a reward varied and was always selected and brought to the test by the owner, who was advised to select the best possible treat for that particular dog. Owners were also advised not to feed the dog prior to the test. However, the amount of time since the dog last ate varied between dogs.

### 2.3. Cylinder Test

The cylinder test was used to assess the dog’s level of inhibitory control. In this task, the dog can see a food treat inside a transparent cylinder. To get access to the reward the dog needs to go around the front of the cylinder to one of the sides and inhibit reaching for the visible reward directly [16,17,18,19,20,23].

**Figure 1:**
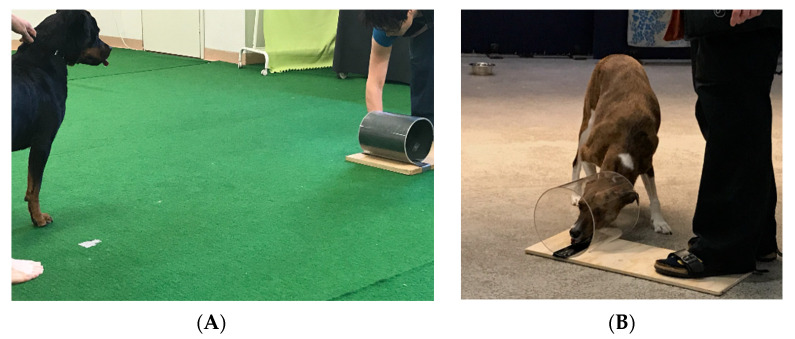
Test set-up for the cylinder test. (**A**) Opaque cylinder during training trials. The dog is waiting to be released before starting the trial. (**B**) Transparent cylinder during test trials. The dog is going around the transparent barrier to access the treat inside the cylinder.

The apparatus consisted of an acrylic cylinder (25 cm in length, 20 cm in diameter) that was open on both sides and attached to a wooden base for support. During trials, the experimenter held the wooden base in place with her foot. In training trials, the cylinder was made opaque by placing a dark piece of cardboard inside, while, in test trials, the cylinder was made transparent by removing the cardboard (Figure 1).

The experiment began with a series of training trials to introduce the dog to the solution to the task. The owner positioned the dog at the start line, 2–2.5 m from the apparatus and held onto its collar. The experimenter showed the dog a food treat while attracting the dog’s attention by calling its name. The experimenter then placed the treat in the middle of the tube, making it accessible to the dog via either side. The experimenter then indicated to the owner that they should release the dog. The owner and experimenter remained standing quietly in place without interacting with the dog, while the dog ate the treat from inside the cylinder. After this, the owner took the dog back to the start line and held it again by its collar while the experimenter placed another treat inside the cylinder.

After each trial, the experimenter recorded whether the dog made a correct or incorrect choice. A choice was coded as “correct” if the dog’s snout entered either of the open ends of the cylinder without the dog first touching the exterior of the cylinder with its snout or paw. Conversely, a choice was coded as “incorrect” if the dog touched the exterior of the cylinder with its snout or paw prior to recovering the treat. In both scenarios, the dog was allowed to eat the treat. Nonchoice responses were coded as “incorrect”. In order to move from the training phase on to the test trials, the dog was required to make four out of five correct choices. The maximum trial number in the training phase was set to 15.

The 10 test trials were identical to the training trials except that the cylinder was transparent. As in training trials, the experimenter recorded whether the dog made a correct or incorrect choice on each trial. The percentage of successful trials was calculated from these. Success in this task varied from 0% (errors in all ten trials) to 100% (no errors made in ten trials). The total duration of the test was approximately 5–10 min.

### 2.4. Unsolvable Task

The unsolvable task [35] was used to assess the dogs’ tendency to initiate social contact with a human during a difficult problem-solving situation, where the dog is confronted with a task which is impossible to solve. The dog has three opportunities for action in this situation: (*a*) persisting with the problem and attempting to solve it independently, (*b*) seeking attention from a human by using gaze, physical contact, or vocalizations, or (*c*) abandoning the task. This test has been used extensively in canine cognitive research to assess social cognition and persistence in dogs [34,36,38,48,52]. This task included a training phase and a 2 min test phase. The intention of the training phase was to give the dog the impression that the task is solvable, after which the task became impossible to solve.

**Figure 2:**
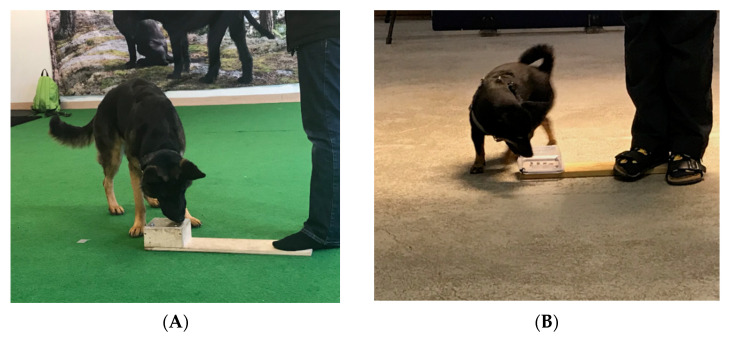
Test setup for the unsolvable task. Images are from the test phase, when the lid is fully closed, and the box is impossible to open. (**A**) Wooden box used for large dogs. (**B**) Plastic box used for small dogs.

The device for the unsolvable task consisted of a box, which had a transparent lid with several small holes to allow the dog to smell the food placed inside. The box for large dogs was wooden (15 cm × 15 cm × 6 cm, 15 cm × 15 cm × 9 cm, or 20 cm × 20 cm × 9 cm), whereas small-sized dogs were provided with a plastic box (11.5 cm × 11.5 cm × 7 cm) (Figure 2). The box was placed on the floor and attached to a wooden base for support. During trials the experimenter held the wooden base in place with her foot, to prevent the dog from moving the box while trying to open it. The experimenter stood next to the box.

**Figure 3:**
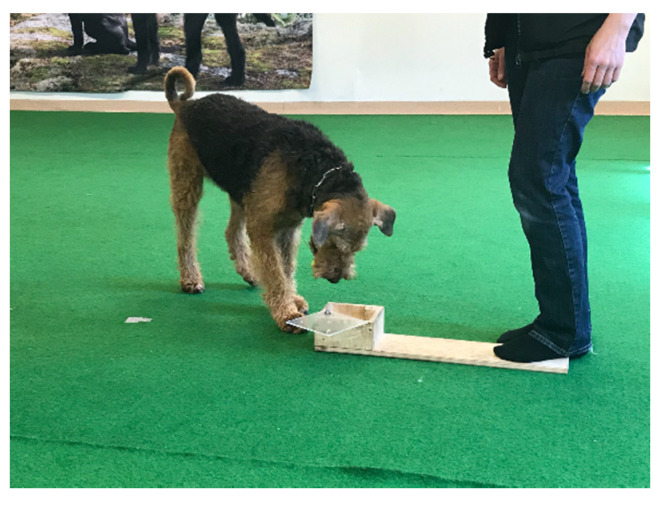
Test setup during the training phase of the unsolvable task. The lid is open, allowing the dog to easily access it. The box was then placed in front of the dog again, and the dog was released. For 2 min, the dog was allowed to move freely around the room and manipulate the box. The owner was advised to stay quiet and only look at the box, and the experimenter did the same.

During the training trials, the dog was first taught to eat treats from the box with the lid taken off (Figure 3). While the owner held the dog by its collar 1–1.5 m away, the experimenter placed a treat (or alternatively a toy) inside the box while attracting the dog’s attention by talking. The experimenter then gave permission to the owner to release the dog. In the training phase, both the experimenter and the owner encouraged the dog to eat the treat from the box. Once the dog had eaten the treat, the owner retrieved the dog and held it again by its collar while the experimenter placed another treat inside the box. In these training trials (*N* = 4), the box was easy to open, and the lid was closed gradually more in each trial, making it slightly more difficult for the dog to open the lid.

During the subsequent 2 min period, two (silent) stopwatches were used to keep track of the amount of time the dog spent engaging in independent versus social behavior. The dog’s behavior was coded as “independent” if the dog manipulated the box with its nose or paw. Behavior was coded as “social” if the dog gazed at the owner/experimenter (either a short glance or a longer period of looking at the human), alternated its gaze between the box and the owner/experimenter, vocalized while looking at the owner/experimenter, or tried some other previously learned tasks, such as lying down or sitting while looking at the owner/experimenter. The dog was considered to have abandoned the task if it engaged in any other behaviors not coded as “independent” or “social”. This may have involved walking away from the box, looking away from the box and the humans, sniffing the ground, or licking itself. At the end of the 2 min period, the experimenter recorded the number of seconds spent on each of the three behaviors during the test phase.

### 2.5. Data Analysis

All statistical analyses were performed using IBM SPSS Statistics Version 25. Data were checked for normality, both visually and using the Kolmogorov–Smirnov test. As the data were not distributed normally (*p* < 0.001), nonparametric tests were used.

Mann–Whitney U-tests were used to compare test results between males and females, and between neutered and intact dogs (including both sexes). Kruskal–Wallis tests were used to analyze differences among neutered males, neutered females, intact males, and intact females in their results for the unsolvable task and cylinder test. Post hoc Mann–Whitney U-tests were used to analyze differences between the following pair comparisons: neutered and intact dogs, neutered males and intact males, spayed females and intact females, neutered males and neutered females, and intact males and intact females.

In addition, we analyzed whether a correlation exists between age and performance in the unsolvable task using Spearman’s rank correlation, since previous studies have shown that increasing age may improve human-directed behavior during the unsolvable task [40,41].

## 3. Results

### 3.1. Cylinder Test

When all individuals were included in the analysis (both neutered and intact), females had a significantly higher success rate in the cylinder test compared to males (U = 102,501.5, *p* < 0.001), indicating that males behaved more impulsively in the task. The success rate of males was 69.9% (SD = 24.3), and, for females, it was 76.8% (SD = 21.5). Reproductive status did not have an effect on test results, when including both females and males in the analysis.

**Figure 4:**
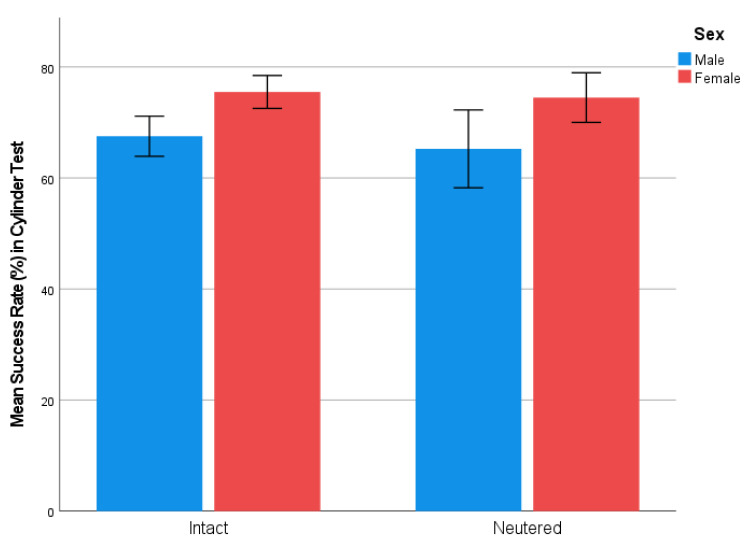
Mean success rates (%) of intact males (*n* = 189), intact females (*n* = 197), neutered males (*n* = 61), and neutered (*n* = 92) in the cylinder test (95% confidence interval).

The same result was found when comparing only intact individuals. Intact females had a significantly higher success rate in the cylinder test compared to intact males (U = 15,112.5, *p* = 0.001) (Figure 4). Similarly, neutered females had a higher success rate than neutered males, although this difference was not statistically significant (U = 2295, *p* = 0.053) (Figure 4). There were no significant differences between neutered males and intact males or between neutered females and intact females. These results suggest that neutering status did not have an effect on performance in the cylinder task.

### 3.2. Unsolvable Task

There was no significant correlation between age (all dogs were >12 months) and time spent on the independent strategy or the social strategy in the unsolvable task.

**Figure 5:**
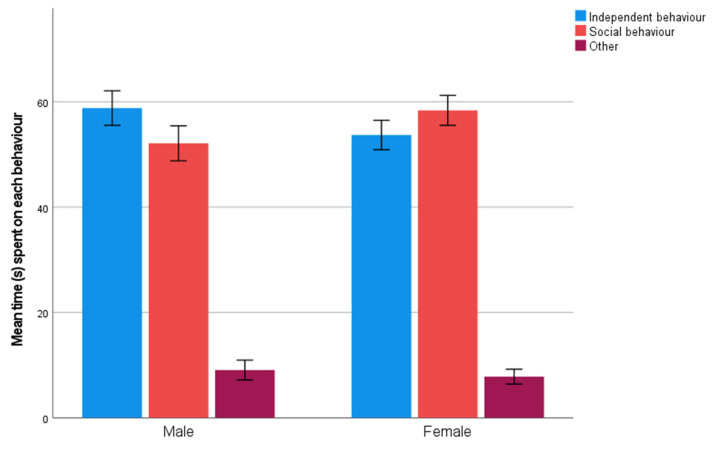
Mean time (s) spent on each behavioral strategy (independent, social, or abandoning the task) for males (*n* = 468) and females (*n* = 539) during the unsolvable task (95% confidence interval).

Females spent significantly more time on social behavior during the unsolvable task compared to males (U = 111,995, *p* = 0.005) (Figure 5). Males spent significantly more time on independent behavior during the unsolvable task compared to females (U = 115,109, *p* = 0.036) (Figure 5). Sex had no significant effect on the amount of time spent on other behaviors (i.e., abandoning the task) (U = 123,302.5, *p* = 0.729) (Figure 5).

Neutered and intact dogs (when including both females and males in the analysis) did not significantly differ from each other in their time spent on independent behavior (*p* = 0.847), social behavior (*p* = 0.751), or abandoning the task (*p* = 0.412). There were no significant differences among neutered males, intact males, neutered females, and intact females in time spent on independent behavior (*p* = 0.789), social behavior (*p* = 0.245), or abandoning the task (*p* = 0.312) (Kruskal–Wallis test). Thus, reproductive status did not seem to have a significant effect on time spent on different behaviors during the unsolvable task.

## 4. Discussion

In this study, we found that male dogs were more impulsive than females when assessed using the cylinder test. Males had a significantly higher error rate compared to females, reflecting greater impulsive action in males. This contrasts with results from previous canine studies, which have found no differences between sexes in impulsive action, whether using the cylinder test or other measures [17,19,27]. However, our results are in line with most research conducted on other mammalian species, since impulsive action has often been observed to be higher in males [24].

There is likely to be an evolutionary basis for these sex differences in certain species. Males may be hypersensitive to rewards and hyposensitive to punishment, whereas females might be more sensitive to punishment and hyposensitive to rewards [53]. Males’ reproductive success often depends on competition for mates and ability to hunt; therefore, they might be more risk-seeking—a trait often linked to impulsivity. In contrast, females may need to exhibit greater inhibitory control because their reproductive success often depends on avoiding harm to themselves and their offspring. The energy expenditure for females is often greater, and they may need to prioritize the requirements of their offspring over their own. Inhibitory control may also be required for optimal mate selection in females, whereas, for males, it may be less advantageous to inhibit their approach behavior [54].

It is possible that the small sample sizes and the limited number of breeds included in previous canine studies have affected earlier results. The specific breeds included could have a substantial effect on results, as there are most likely breed differences in impulsivity [28]. Impulsivity has also recently been shown to be a highly heritable trait in dogs [9]. Tiira and colleagues [23] found that police explosive search dogs (mostly working lines of Malinois, German Shepherd, and Labrador Retriever) had an average success rate of 66.8% in the cylinder task, compared to mean success rates of studies conducted in pet dogs of various breeds (82% [20] and 95% [18]). Thus, if sample sizes are small and breed variation is uncontrolled, this could easily result in mixed findings, depending on the breed composition of different sex groups.

Inhibitory control has been measured in humans [55], nonhuman animals [16,56], and dogs [57] using various approaches, such as behavior tests and questionnaires. It seems that, in most species, different measures do not correlate with each other and likely measure different aspects of inhibitory control [17,19,20,57]. Furthermore, tasks are most likely context-specific [20]. Studies investigating the effect of sex on inhibitory control have also used various methodologies. For example, in Fagnani and colleagues’ [19] study, if dogs made an incorrect response, the dog was told “no” and was not allowed to eat the treat. In the current study, dogs were allowed to freely interact with the apparatus and eat the treat regardless of their behavior during the test. Similarly, DIAS questionnaires may be more reflective of impulsive choice rather than impulsive action [13].

Our results showed that female dogs spent a significantly longer time behaving socially toward a human during the unsolvable task compared to males, which spent significantly longer attempting to solve the problem independently. Our results are consistent with those found in previous studies investigating dog behavior during an unsolvable task [40,41], although one study [39] did not replicate these findings.

The discrepancy among the studies could be due to methodological differences. The study by Konno and colleagues [39] measured the dogs’ behavior for 1 min (compared to 2 min in our study), and the owner was not present in the room. According to previous findings, both differences in methodology could have affected the results [34].

Our results are further supported by the observation that female dogs tend to spend more time looking at humans in various situations [43,44,45,46,47,58], and they are also more likely to rely on visual information to solve problems [5,48]. These findings are also in line with results from similar studies in other species. Several studies report that women have more interest in social information, are better at facial expression recognition, and understand gestures and body language better [59]. In macaques, females are more likely to follow the gaze of conspecifics than males [60]. Sex-specific differences in social behavior depend on the ecology and evolutionary history of a specific species. In many mammalian species, males compete over access to females, making aggressive behavior potentially more adaptive [61]. In contrast, females may prioritize other resources which improve their reproductive success, such as positive social contacts (especially in social species).

In our study, females were more likely to engage in social behavior/cooperation when faced with a problem, possibly attempting to request help from the human. The other possible conclusion is that males are more persistent than females in problem-solving situations. Previous research has provided mixed results on whether the unsolvable task assesses social communication or persistence [23,34,36,62,63]. However, recent studies have found that dogs which scored higher in human-directed gazing were more likely to be selected as service or detection dogs in the future [64,65]. In addition, field-type Labrador Retrievers were found to gaze more at humans compared to pet and show Labrador Retrievers [66]. These results suggest that human-directed behavior during the unsolvable task may indicate more willingness for cooperation. 

No effect of reproductive status on the dogs’ cognitive traits was seen in this study. Neutered dogs did not significantly differ from intact dogs in the cylinder test or the unsolvable task. Similar results were found by Duranton and colleagues [46]; neutering had no effect on the amount of human-directed eye contact. In contrast, Mongillo and colleagues [47] found that intact females spent the longest time gazing at people, and Scandurra and colleagues [49] found that intact females outperformed spayed females in their socio-cognitive abilities. 

These contrasting findings may have arisen due to different neutering practices in different countries. Our study included three times more intact males than castrated males and two times more intact females compared to spayed females. Neutering is considerably less common in Finland than in many other countries, where dogs may be routinely neutered as a precaution. Most importantly, dogs are neutered much later in life in Finland compared to many other countries. For example, in the United States, dogs can be neutered at 6–14 weeks at the youngest [67], whereas, in Finland, it is more common to neuter dogs in adulthood (if at all). Later neutering means that reproductive hormones are able to affect the dog for a longer time, and behaviors may become more permanent. Previous studies show that age at neutering can affect resulting adult behavior, such as aggression and fearfulness [68,69].

Neutering of dogs is not routinely done, nor is it random in Finland, and some individuals are more likely to be neutered than others. For example, rescue dogs are routinely neutered before adoption, whereas pedigree dogs may be more likely to remain intact, especially if they participate in dog shows or competitions. Dogs are also occasionally neutered due to problem behaviors, such as male aggression. Some of these behavior problems (e.g., aggression) may also be linked to impulsivity or persistence [10,11,12,14,70,71], which may further complicate the picture.

Different methodologies may also partly explain the contrasting results. Fadel and colleagues [28] found that neutered males were more impulsive than intact males. This study differed largely in methodology from the current study, since it was conducted via owner-filled questionnaire (DIAS). It is possible that results from the DIAS questionnaire are more indicative of impulsive choice rather than impulsive action [13], which could explain the different findings.

Since reproductive status did not seem to affect the results of either test, it is likely that differences observed between sexes resulted mainly through chromosome effects or organizational effects during early development. According to our results, circulating levels of reproductive hormones are unlikely to have had a large effect on behavior in these specific tests.

It is, however, important to note that we did not directly measure the level of circulating reproductive hormones of individual dogs, and more detailed studies are needed to verify the exact effect of hormonal levels on cognitive traits. Female dogs may have been at different stages of their ovarian cycles, which could have affected hormone levels. Furthermore, baseline testosterone and estrogen levels may show notable variation between individuals [72,73,74].

It is also a possibility that the sex differences found in impulsivity and social cognition in our study are influenced by some other personality trait which differs between males and females, such as boldness or anxiety. The shyness–boldness continuum is considered one of the fundamental personality axes in humans and other animals, including dogs [75]. ‘Boldness’ is usually associated with assertiveness, exploratory activity, risk-taking, and interest in novel environments, objects, and individuals, whereas ‘shyness’ tends to be associated with cautiousness, timidity, and fear of novelty. Male dogs are generally bolder than females [76,77], and boldness can have an effect on various behavioral and cognitive traits in dogs and other species [78,79,80,81]. According to several studies, female dogs are also more likely to be fearful or anxious than males [29], and anxiety and stress might also affect performance in cognitive tasks [82,83].

The gender of the experimenter could also be a contributing factor, since male and female dogs may show a different behavioral response to men compared to women [37,43,84]. However, as the tests were always performed by a woman and 95.3% of the owners in our data were female, it was not possible to investigate the effect of human gender on test performance of the dogs. Our results may, therefore, be indicative of dog behavior in the presence of a female owner and experimenter, and future studies could investigate whether test performance is affected by owner gender.

To the best of our knowledge, our study used the largest experimental dataset on canine cognition so far—with the exception of citizen science projects, where owners perform the tests themselves at home [9], whereas our study was more standardized and controlled. The range of breeds in our study was also exceptionally large compared to other similar studies.

Understanding behavioral and cognitive differences between male and female dogs may help with choosing suitable individuals for their working roles. According to our results, males may be more suited to roles which require high impulsivity and independence, whereas females may be the ideal choice for roles requiring high inhibitory control and cooperation with humans. Nevertheless, it is good to keep in mind that both traits—inhibitory control and social cognition (or cooperation)—have a significant heritable component. Selective breeding for working roles requiring either high arousal or high cooperativeness has resulted in differences in these traits in different breeding lines.

## 5. Conclusions

Our results show that, compared to males, female dogs had higher inhibitory control and were more likely to gaze at humans during a problem-solving situation. In contrast, males were more likely to resort to impulsive action, and they tended to engage in a more independent strategy when solving a problem. Reproductive status did not have a significant effect on results in the cylinder test or the unsolvable task; therefore, circulating levels of sex hormones were unlikely to have affected dogs’ behavior during these tests. These findings help to provide a more complete picture of behavioral and cognitive sex differences in dogs.

## Figures and Tables

**Table 1:** Mean ages and number of dogs included in the analysis, divided by sex and reproductive status.

Sex and Reproductive Status	No. of Dogs with Reproductive Status Known	No. of Dogs with Sex Known	Mean Age
Intact dogs	392		3.7
Neutered dogs	155		5.3
Males	255	483	4.1
Intact	198		
Neutered	61		
Females	292	549	4.2
Intact	198		
Neutered	94		
All dogs	547	1032	4.2

## Data Availability

The data presented in this study are available on request from the corresponding authors due to privacy restrictions.

## Appendix A

**Table A1:** Number of individuals from each breed included in the study.

Breed	Total Number	Males	Females
Airedale Terrier	2	2	0
Akita	1	0	1
Alaskan Malamute	3	1	2
American Hairless Terrier	13	3	10
American Staffordshire Terrier	3	1	2
Appenzeller Sennenhund	3	3	0
Australian Cattle Dog	2	1	1
Australian Kelpie	24	13	11
Australian Shepherd	23	12	11
Australian Silky Terrier	1	1	0
Australian Terrier	2	1	1
Auvergne Pointer	1	0	1
Barbet	2	2	0
Basset Fauve de Bretagne	1	0	1
Beagle	5	2	3
Bearded Collie	7	3	4
Beauceron	4	0	4
Belgian Shepherd	44	28	16
Bernese Mountain Dog	8	2	6
Bichon Frisé	1	0	1
Black Russian Terrier	3	2	1
Bloodhound	1	1	0
Bohemian Shepherd	4	1	3
Bolognese	3	1	2
Border Collie	60	24	36
Border Terrier	11	3	9
Boston Terrier	4	2	2
Bouvier	3	1	2
Boxer	2	0	2
Braque du Bourbonnais	2	1	1
Broholmer	2	2	0
Bull Mastiff	5	2	3
Bull Terrier	1	0	1
Cairn Terrier	2	0	2
Cane Corso	2	2	0
Cavalier King Charles Spaniel	3	2	1
Ceskoslovenski Vlcak	2	1	1
Chesapeake Bay Retriever	1	1	0
Chihuahua	1	1	0
Chinese Crested Dog	2	0	2
Cocker Spaniel	30	14	16
Coton de Tulear	1	1	0
Croatian Sheepdog	1	0	1
Curly-Coated Retriever	4	2	2
Dalmatian	3	2	1
Danish-Swedish Farmdog	25	17	8
Dutch Shepherd	7	2	5
East European Shepherd	2	1	1
English Springer Spaniel	6	5	1
English Toy Terrier	2	1	1
Eurasier	1	0	1
Field Spaniel	3	1	2
Finnish Lapphund	28	17	11
Finnish Spitz	5	1	4
Flat-Coated Retriever	28	14	14
Fox Terrier	1	0	1
German Pointer	4	1	3
German Shepherd	41	21	20
Giant Schnauzer	18	10	8
Glen of Imaal Terrier	1	1	0
Golden Retriever	21	6	15
Great Dane	2	1	1
Hovawart	23	11	12
Hungarian Vizsla	5	1	4
Icelandic Sheepdog	2	1	1
Irish Setter	4	4	0
Irish Terrier	3	0	3
Irish Wolfhound	2	1	1
Italian Greyhound	2	0	2
Jack Russell Terrier	9	4	5
Karelian Bear Dog	1	1	0
Keeshond	1	0	1
Kerry Blue Terrier	4	2	2
Kleinspitz	3	0	3
Kooikerhondje	6	4	2
Labrador Retriever	59	26	33
Lagotto Romagnolo	8	5	3
Lancashire Heeler	5	3	2
Landseer	1	1	0
Lapponian Herder	17	10	7
Leonberger	5	3	2
Manchester Terrier	1	0	1
Medium Poodle	6	3	3
Miniature Daschund	12	3	9
Miniature Pinscher	3	2	1
Miniature Schnauzer	5	2	3
Mittelspitz	9	7	2
Mixed Breed	90	27	63
Mudi	12	8	4
Nova Scotia Duck Tolling Retriever	15	4	11
Parson Russell Terrier	7	4	3
Petit Brabacon	2	2	0
Pinscher	3	3	0
Portuguese Podengo	5	1	4
Portuguese Sheepdog	1	1	0
Portuguese Water Dog	2	1	1
Pumi	2	2	0
Pyrenean Sheepdog	5	2	3
Rhodesian Ridgeback	10	2	8
Rottweiler	10	3	7
Rough Collie	10	5	5
Russian Hound	1	0	1
Russian Tsvetnaya Bolonka	1	1	0
Saluki	14	6	8
Samoyed	1	1	0
Schapendoes	3	3	0
Schipperke	2	0	2
Schnauzer	2	1	1
Seiskarinkoira	1	0	1
Shetland Sheepdog	16	8	8
Shikoku	4	2	2
Siberian Husky	1	0	1
Silken Windhound	1	0	1
Small Munsterlander	1	1	0
Smooth Collie	6	2	4
Spanish Galgo	2	1	1
Spanish Water Dog	29	9	20
Stabyhoun	1	1	0
Staffordshire Bull Terrier	4	2	2
Standard Poodle	7	1	6
Swedish Vallhund	19	13	6
Tibetan Mastiff	3	1	2
Tibetan Spaniel	3	0	3
Weimaraner	2	1	1
Welsh Corgi	4	2	2
Welsh Springer Spaniel	7	4	3
West Highland White Terrier	7	4	3
Wheaten Terrier	3	1	2
Whippet	2	1	1
White Shepherd	7	4	3
Yorkshire Terrier	1	1	0
Not reported	9	6	3

## References

[1] Jacobs L.F., Gaulin S.J., Sherry D.F., Hoffman G.E. (1990). Evolution of spatial cognition: Sex-specific patterns of spatial behavior predict hippocampal size. Proc. Natl. Acad. Sci. USA.

[2] Locklear M.N., Kritzer M.F. (2014). Assessment of the effects of sex and sex hormones on spatial cognition in adult rats using the Barnes maze. Horm. Behav..

[3] Bachevalier J., Hagger C. (1991). Sex differences in the development of learning abilities in primates. Psychoneuroendocrinology.

[4] Müller C.A., Mayer C., Dörrenberg S., Huber L., Range F. (2011). Female but not male dogs respond to a size constancy violation. Biol. Lett..

[5] Fugazza C., Mongillo P., Marinelli L. (2017). Sex differences in dogs’ social learning of spatial information. Anim. Cogn..

[6] Mongillo P., Scandurra A., D’Aniello B., Marinelli L. (2017). Effect of sex and gonadectomy on dogs’ spatial performance. Appl. Anim. Behav. Sci..

[7] Scandurra A., Pinelli C., Fierro B., Di Cosmo A., D’Aniello B. (2020). Multimodal signaling in the visuo-acoustic mismatch paradigm: Similarities between dogs and children in the communicative approach. Anim. Cogn..

[8] Arnold A.P. (2009). The organizational–activational hypothesis as the foundation for a unified theory of sexual differentiation of all mammalian tissues. Horm. Behav..

[9] Gnanadesikan G.E., Hare B., Snyder-Mackler N., MacLean E.L. (2020). Estimating the heritability of cognitive traits across dog breeds reveals highly heritable inhibitory control and communication factors. Anim. Cogn..

[10] Fatjo J., Amat M., Mariotti V.M., de la Torre J.L.R., Manteca X. (2007). Analysis of 1040 cases of canine aggression in a referral practice in Spain. J. Vet. Behav..

[11] Vas J., Topál J., Péch É., Miklósi Á. (2007). Measuring attention deficit and activity in dogs: A new application and validation of a human ADHD questionnaire. Appl. Anim. Behav. Sci..

[12] Amat M., Manteca X., Mariotti V.M., de la Torre J.L.R., Fatjó J. (2009). Aggressive behavior in the English cocker spaniel. J. Vet. Behav..

[13] Wright H.F., Mills D.S., Pollux P.M.J. (2012). Behavioural and physiological correlates of impulsivity in the domestic dog (*Canis familiaris*). Physiol. Behav..

[14] Piotti P., Satchell L.P., Lockhart T.S. (2018). Impulsivity and behaviour problems in dogs: A Reinforcement Sensitivity Theory perspective. Behav. Process..

[15] Winstanley C.A., Eagle D.M., Robbins T.W. (2006). Behavioral models of impulsivity in relation to ADHD: Translation between clinical and preclinical studies. Clin. Psychol. Rev..

[16] MacLean E.L., Hare B., Nunn C.L., Addessi E., Amici F., Anderson R.C., Aureli F., Baker J.M., Bania A.E., Barnard A.M. (2014). The evolution of self-control. Proc. Natl. Acad. Sci. USA.

[17] Bray E.E., Bray E.E., MacLean E.L., MacLean E.L., Hare B.A., Hare B.A. (2014). Context specificity of inhibitory control in dogs. Anim. Cogn..

[18] Marshall-Pescini S., Virányi Z., Range F. (2015). The Effect of Domestication on Inhibitory Control: Wolves and Dogs Compared. PLoS ONE.

[19] Fagnani J., Barrera G., Carballo F., Bentosela M. (2016). Is previous experience important for inhibitory control? A comparison between shelter and pet dogs in A-not-B and cylinder tasks. Anim. Cogn..

[20] Vernouillet A.A.A., Stiles L.R., Andrew McCausland J., Kelly D.M. (2018). Individual performance across motoric self-regulation tasks are not correlated for pet dogs. Learn. Behav..

[21] Kabadayi C., Bobrowicz K., Osvath M. (2018). The detour paradigm in animal cognition. Anim. Cogn..

[22] Krichbaum S., Lazarowski L., Smith J.G., Cox E., Katz J.S., Mills D.S., Matos R., Jacinto D. (2021). Assessing Inhibitory Control in Dogs Is No Easy Task: Factors to Consider, Canine Science Forum, 6–9 July 2021.

[23] Tiira K., Tikkanen A., Vainio O. (2020). Inhibitory control—Important trait for explosive detection performance in police dogs?. Appl. Anim. Behav. Sci..

[24] Weafer J., de Wit H. (2014). Sex differences in impulsive action and impulsive choice. Addict. Behav..

[25] Gaillard A., Fehring D.J., Rossell S.L. (2021). A systematic review and meta-analysis of behavioural sex differences in executive control. Eur. J. Neurosci..

[26] Mongillo P., Scandurra A., Eatherington C.J., D’Aniello B., Marinelli L. (2019). Development of a Spatial Discount Task to Measure Impulsive Choices in Dogs. Animals.

[27] Lazarowski L., Krichbaum S., Waggoner L.P., Katz J.S. (2020). The development of problem-solving abilities in a population of candidate detection dogs (*Canis familiaris*). Anim. Cogn..

[28] Fadel F.R., Driscoll P., Pilot M., Wright H., Zulch H., Mills D. (2016). Differences in Trait Impulsivity Indicate Diversification of Dog Breeds into Working and Show Lines. Sci. Rep..

[29] Salonen M., Sulkama S., Mikkola S., Puurunen J., Hakanen E., Tiira K., Araujo C., Lohi H. (2020). Prevalence, comorbidity, and breed differences in canine anxiety in 13,700 Finnish pet dogs. Sci. Rep..

[30] Nave G., Nadler A., Zava D., Camerer C. (2017). Single-Dose Testosterone Administration Impairs Cognitive Reflection in Men. Psychol. Sci..

[31] Jentsch J.D., Taylor J.R. (2003). Sex-related differences in spatial divided attention and motor impulsivity in rats. Behav. Neurosci..

[32] Swalve N., Smethells J.R., Carroll M.E. (2016). Progesterone attenuates impulsive action in a Go/No-Go task for sucrose pellets in female and male rats. Horm. Behav..

[33] Hernandez C.M., Orsini C., Wheeler A., Ten Eyck T.W., Betzhold S.M., Labiste C.C., Wright N.G., Setlow B., Bizon J.L. (2020). Testicular hormones mediate robust sex differences in impulsive choice in rats. eLife.

[34] Mendes J.W.W., Resende B., Savalli C. (2021). A review of the unsolvable task in dog communication and cognition: Comparing different methodologies. Anim. Cogn..

[35] Miklósi Á., Kubinyi E., Topál J., Gácsi M., Virányi Z., Csányi V. (2003). A Simple Reason for a Big Difference: Wolves Do Not Look Back at Humans, but Dogs Do. Curr. Biol..

[36] Marshall-Pescini S., Rao A., Virányi Z., Range F. (2017). The role of domestication and experience in ‘looking back’ towards humans in an unsolvable task. Sci. Rep..

[37] Carballo F., Cavalli C., Martínez M., Dzik V., Bentosela M. (2020). Asking for help: Do dogs take into account prior experiences with people?. Learn. Behav..

[38] Cavalli C., Carballo F., Dzik M.V., Bentosela M. (2020). Gazing as a help requesting behavior: A comparison of dogs participating in animal-assisted interventions and pet dogs. Anim. Cogn..

[39] Konno A., Romero T., Inoue-Murayama M., Saito A., Hasegawa T. (2016). Dog Breed Differences in Visual Communication with Humans. PLoS ONE.

[40] Hori Y., Kishi H., Inoue-Murayama M., Fujita K. (2013). Dopamine receptor D4 gene (DRD4) is associated with gazing toward humans in domestic dogs (*Canis familiaris*). Open J. Anim. Sci..

[41] Persson M.E., Roth L.S.V., Johnsson M., Wright D., Jensen P. (2015). Human-directed social behaviour in dogs shows significant heritability. Genes Brain Behav..

[42] Carballo F., Cavalli C.M., Gácsi M., Miklósi Á., Kubinyi E. (2020). Assistance and Therapy Dogs Are Better Problem Solvers Than Both Trained and Untrained Family Dogs. Front. Vet. Sci..

[43] Lore R.K., Eisenberg F.B. (1986). Avoidance reactions of domestic dogs to unfamiliar male and female humans in a kennel setting. Appl. Anim. Behav. Sci..

[44] Foyer P., Wilsson E., Wright D., Jensen P. (2013). Early experiences modulate stress coping in a population of German shepherd dogs. Appl. Anim. Behav. Sci..

[45] Nagasawa M., Mitsui S., En S., Ohtani N., Ohta M., Sakuma Y., Onaka T., Mogi K., Kikusui T. (2015). Oxytocin-gaze positive loop and the coevolution of human-dog bonds. Science.

[46] Duranton C., Bedossa T., Gaunet F. (2016). When facing an unfamiliar person, pet dogs present social referencing based on their owners’ direction of movement alone. Anim. Behav..

[47] Mongillo P., Pitteri E., Candaten M., Marinelli L. (2016). Can attention be taught? Interspecific attention by dogs (*Canis familiaris*) performing obedience tasks. Appl. Anim. Behav. Sci..

[48] D’Aniello B., Scandurra A. (2016). Ontogenetic effects on gazing behaviour: A case study of kennel dogs (Labrador Retrievers) in the impossible task paradigm. Anim. Cogn..

[49] Scandurra A., Alterisio A., Di Cosmo A., D’Ambrosio A., D’Aniello B. (2019). Ovariectomy Impairs Socio-Cognitive Functions in Dogs. Animals.

[50] SmartDOG Oy. https://www.smartdog.fi/english.

[51] Passalacqua C., Marshall-Pescini S., Barnard S., Lakatos G., Valsecchi P., Prato Previde E. (2011). Human-directed gazing behaviour in puppies and adult dogs, Canis lupus familiaris. Anim. Behav..

[52] Marshall-Pescini S., Colombo E., Passalacqua C., Merola I., Prato-Previde E. (2013). Gaze alternation in dogs and toddlers in an unsolvable task: Evidence of an audience effect. Anim. Cogn..

[53] Orsini C.A., Setlow B. (2017). Sex differences in animal models of decision making. J. Neurosci. Res..

[54] Hosseini-Kamkar N., Morton J.B. (2014). Sex differences in self-regulation: An evolutionary perspective. Front. Neurosci..

[55] Duckworth A.L., Kern M.L. (2011). A meta-analysis of the convergent validity of self-control measures. J. Res. Personal..

[56] Mayse J.D., Nelson G.M., Park P., Gallagher M., Lin S. (2014). Proactive and reactive inhibitory control in rats. Front. Neurosci.

[57] Brucks D., Marshall-Pescini S., Wallis L.J., Huber L., Range F. (2017). Measures of Dogs’ Inhibitory Control Abilities Do Not Correlate across Tasks. Front. Psychol..

[58] Foyer P., Bjällerhag N., Wilsson E., Jensen P. (2014). Behaviour and experiences of dogs during the first year of life predict the outcome in a later temperament test. Appl. Anim. Behav. Sci..

[59] Proverbio A.M. (2021). Sex differences in the social brain and in social cognition. J. Neurosci. Res..

[60] Paukner A., Anderson J.R., Fogassi L., Ferrari P.F. (2007). Do facial gestures, visibility or speed of movement influence gaze following responses in pigtail macaques?. Primates.

[61] Archer J. (1996). Sex Differences in Social Behavior: Are the Social Role and Evolutionary Explanations Compatible?. Am. Psychol..

[62] Lazzaroni M., Marshall-Pescini S., Manzenreiter H., Gosch S., Přibilová L., Darc L., McGetrick J., Range F. (2020). Why do dogs look back at the human in an impossible task? Looking back behaviour may be over-interpreted. Anim. Cogn..

[63] Rao A., Bernasconi L., Lazzaroni M., Marshall-Pescini S., Range F. (2018). Differences in persistence between dogs and wolves in an unsolvable task in the absence of humans. PeerJ.

[64] MacLean E.L., Hare B. (2018). Enhanced Selection of Assistance and Explosive Detection Dogs Using Cognitive Measures. Front. Vet. Sci..

[65] Lazarowski L., Strassberg L.R., Waggoner L.P., Katz J.S. (2019). Persistence and human-directed behavior in detection dogs: Ontogenetic development and relationships to working dog success. Appl. Anim. Behav. Sci..

[66] Sundman A., Persson M.E., Grozelier A., Halldén L., Jensen P., Roth L.S.V. (2018). Understanding of human referential gestures is not correlated to human-directed social behaviour in Labrador retrievers and German shepherd dogs. Appl. Anim. Behav. Sci..

[67] Kustritz M.V.R. (2002). Early spay-neuter: Clinical considerations. Clin. Tech. Small Anim. Pract..

[68] Farhoody P., Mallawaarachchi I., Tarwater P.M., Serpell J.A., Duffy D.L., Zink C. (2018). Aggression toward Familiar People, Strangers, and Conspecifics in Gonadectomized and Intact Dogs. Front. Vet. Sci..

[69] Starling M., Fawcett A., Wilson B., Serpell J., McGreevy P. (2019). Behavioural risks in female dogs with minimal lifetime exposure to gonadal hormones. PLoS ONE.

[70] Wright H.F., Mills D.S., Pollux P.M.J. (2011). Development and Validation of a Psychometric Tool for Assessing Impulsivity in the Domestic Dog (*Canis familiaris*). Int. J. Comp. Psychol..

[71] Protopopova A., Hall N.J., Wynne C.D.L. (2014). Association between increased behavioral persistence and stereotypy in the pet dog. Behav. Process..

[72] Vanderstichel R., Forzán M.J., Pérez G.E., Serpell J.A., Garde E. (2015). Changes in blood testosterone concentrations after surgical and chemical sterilization of male free-roaming dogs in southern Chile. Theriogenology.

[73] Frank L.A., Mullins R., Rohrbach B.W. (2010). Variability of estradiol concentration in normal dogs. Vet. Dermatol..

[74] Thun R., Eggenberger E., Zerobin K. (1990). 24-hour profiles of plasma cortisol and testosterone in the male dog: Absence of circadian rhythmicity, seasonal influence and hormonal inter-relationships. Reprod. Domest. Anim..

[75] Svartberg K. (2005). A comparison of behaviour in test and in everyday life: Evidence of three consistent boldness-related personality traits in dogs. Appl. Anim. Behav. Sci..

[76] Starling M.J., Branson N., Thomson P.C., McGreevy P.D. (2013). Age, sex and reproductive status affect boldness in dogs. Vet. J..

[77] Scandurra A., Alterisio A., Di Cosmo A., D’Aniello B. (2018). Behavioral and Perceptual Differences between Sexes in Dogs: An Overview. Animals.

[78] Svartberg K. (2002). Shyness–boldness predicts performance in working dogs. Appl. Anim. Behav. Sci..

[79] Guillette L.M., Hahn A.H., Hoeschele M., Przyslupski A., Sturdy C.B. (2015). Individual differences in learning speed, performance accuracy and exploratory behaviour in black-capped chickadees. Anim. Cogn..

[80] Mazza V., Eccard J.A., Zaccaroni M., Jacob J., Dammhahn M. (2018). The fast and the flexible: Cognitive style drives individual variation in cognition in a small mammal. Anim. Behav..

[81] Daniel D.K., Bhat A. (2020). Bolder and Brighter? Exploring Correlations Between Personality and Cognitive Abilities Among Individuals Within a Population of Wild Zebrafish, Danio rerio. Front. Behav. Neurosci..

[82] Passalacqua C., Marshall-Pescini S., Merola I., Palestrini C., Previde E.P. (2013). Different problem-solving strategies in dogs diagnosed with anxiety-related disorders and control dogs in an unsolvable task paradigm. Appl. Anim. Behav. Sci..

[83] Overall K.L., Dunham A.E., Scheifele P., Sonstrom Malowski K. (2019). Fear of noises affects canine problem solving behavior and locomotion in standardized cognitive tests. Appl. Anim. Behav. Sci..

[84] Wells D.L., Hepper P.G. (1999). Male and female dogs respond differently to men and women. Appl. Anim. Behav. Sci..

